# Discrepancies in Composition and Biological Effects of Different Formulations of Chondroitin Sulfate 

**DOI:** 10.3390/molecules20034277

**Published:** 2015-03-06

**Authors:** Johanne Martel-Pelletier, Aina Farran, Eulàlia Montell, Josep Vergés, Jean-Pierre Pelletier

**Affiliations:** 1 Osteoarthritis Research Unit, University of Montreal Hospital Research Centre (CRCHUM), Montreal, QC H2X 0A9, Canada; E-Mail: dr@jppelletier.ca; 2 Unitat de Recerca en Inflamació i Cartílag, Institut Mar d’Investigacions Mèdiques (IMIM), Barcelona 08003, Spain; E-Mail: ainafdc@gmail.com; 3 Pre-Clinical R&D Area, Pharmascience Division, Bioibérica, Barcelona 08029, Spain; E-Mails: lmontell@bioiberica.com (E.M.); jverges@bioiberica.com (J.V.)

**Keywords:** chondroitin sulfate, biological product, animal origin, purity, process, osteoarthritis, joint structure modification, SYSADOA

## Abstract

Osteoarthritis is a common, progressive joint disease, and treatments generally aim for symptomatic improvement. However, SYmptomatic Slow-Acting Drugs in Osteoarthritis (SYSADOAs) not only reduce joint pain, but slow structural disease progression. One such agent is chondroitin sulfate—a complex, heterogeneous polysaccharide. It is extracted from various animal cartilages, thus has a wide range of molecular weights and different amounts and patterns of sulfation. Chondroitin sulfate has an excellent safety profile, and although various meta-analyses have concluded that it has a beneficial effect on symptoms and structure, others have concluded little or no benefit. This may be due, at least partly, to variations in the quality of the chondroitin sulfate used for a particular study. Chondroitin sulfate is available as pharmaceutical- and nutraceutical-grade products, and the latter have great variations in preparation, composition, purity and effects. Moreover, some products contain a negligible amount of chondroitin sulfate and among samples with reasonable amounts, *in vitro* testing showed widely varying effects. Of importance, although some showed anti-inflammatory effects, others demonstrated weak effects, and some instances were even pro-inflammatory. This could be related to contaminants, which depend on the origin, production and purification process. It is therefore vitally important that only pharmaceutical-grade chondroitin sulfate be used for treating osteoarthritis patients.

## 1. Introduction

Osteoarthritis affects over 40 million Europeans [1] and around 30 million Americans [2]. A recent meta-analysis has reported an overall prevalence of 43% for hand, 24% for knee and 11% for hip osteoarthritis [3]. This disease is associated with a reduced quality of life and increased healthcare costs [4]. As one of the risk factors is age, the burden of this disease is set to rise further with the aging population [1,2]. Treatment goals for osteoarthritis include improvements in physical function, quality of life and reductions in pain and disease progression [4]. Current treatment recommendations include pharmacological (e.g., non-steroidal anti-inflammatory drugs) and non-pharmacological (e.g., patient education) options [5,6,7]. These approaches are generally aimed at symptomatic improvement. However, various compounds (e.g., chondroitin sulfate, glucosamine) are currently being investigated for their effects on both symptoms and joint structural degradation [4]. Chondroitin sulfate, a SYmptomatic Slow-Acting Drug in OsteoArthritis (SYSADOA), is currently recommended by the EUropean League Against Rheumatism (EULAR) [5,6] and the European Society for Clinical and Economic aspects of Osteoporosis and Osteoarthritis (ESCEO) [7] in patients with osteoarthritis of the knee [5,7] or hip [6]. Osteoarthritis is a progressive disease of the whole joint, and involves subchondral bone destruction, the formation of osteophytes (bone spurs), synovial inflammation and cartilage loss [8]. Joint tissue degradation is known to occur via a number of pathways including an increase in matrix metalloproteinases (MMP) and pro-inflammatory and catabolic mediators [9].

## 2. Chondroitin Sulfate

Chondroitin sulfate is one of five classes of glycosaminoglycans, the others being heparan sulfate, keratan sulfate, dermatan sulfate and hyaluronic acid [10]. Glycosaminoglycans are long, linear polysaccharides that contain a repeating disaccharide unit consisting of an amino sugar (*N*-acetylglucosamine or *N*-acetylgalactosamine) and a uronic (glucuronic or iduronic) acid or galactose [11]. Various sulfated residues (except in hyaluronic acid) regulate their biological functions [11]. Chondroitin sulfate [4] and hyaluronic acid [12] have been widely used in the treatment of osteoarthritis.

### 2.1. Structure

**Figure 1:**
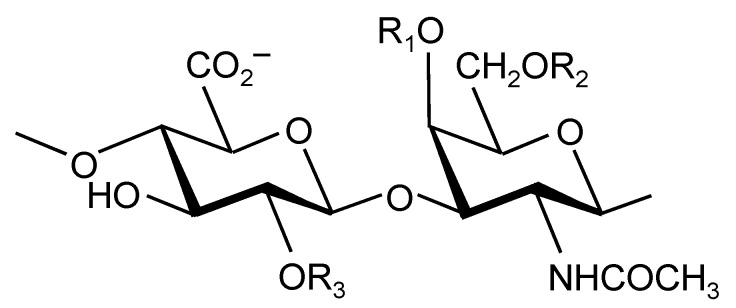
Structures of disaccharides forming chondroitin sulfate. R_1_ = R_2_ = R_3_ = H: non-sulfated chondroitin. R_1_ = SO_3_^−^; R_2_ = R_3_ = H: chondroitin-4-sulfate; R_1_ = R_3_ = SO_3_^−^; R_2_ = H: chondroitin-2,4-disulfate; R_2_ = SO_3_–; R_1_ = R_3_ = H: chondroitin-6-sulfate; R_2_ = R_3_ = SO_3_^−^; R_1_ = H: chondroitin-2,6-disulfate; R_1_ = R_2_ = SO_3_^−^; R_3_ = H: chondroitin-4,6-disulfate; R_1_ = R_2_ = R_3_ = SO_3_^−^: trisulfated chondroitin.

Chondroitin sulfate is a sulfated glycosaminoglycan composed of chains of alternating d-glucuronic acid and *N*-acetyl-d-galactosamine. Chondroitin sulfate disaccharides can have different amounts and patterns of sulfation. Figure 1 shows the most common sulfation patterns, but it is also possible to have sulfation on the free OH [13,14]. Each chondroitin sulfate chain can contain a mixture of disaccharides and be of varying length [15]. Hence, the molecular weight of naturally occurring chondroitin sulfate can vary widely, from 50 to 100 kDa [16]. Chondroitin sulfate is therefore a complex, heterogeneous polysaccharide with varying charge density and molecular weight, which can affect its chemical properties and biological/pharmacological activities [17].

Chondroitin sulfate is not a sulfate salt of chondroitin; rather, the sulfate is covalently bound to the molecule [18]. As such, chondroitin sulfate should more correctly be called sodium chondroitin (as there are ionic bonds between the sulfate groups and sodium ions) [18], but in this review, the more common term chondroitin sulfate is used.

### 2.2. Production

Chondroitin sulfate occurs naturally in the extracellular matrix of connective tissues, e.g., bone, cartilage, skin, ligaments and tendons [19]. Commercially available chondroitin sulfate is extracted from animal cartilage and then purified.

The species (e.g., bovine, porcine, piscine, avian) and tissue (e.g., trachea, ear, nose) affect the molecular composition (sulfation and chain length). The extraction process results in some degradation, reducing the molecular weight from 50–100 kDa to around 10–40 kDa [16]. To minimize chemical and structural modifications during extraction, a selective and robust extraction process should be used; while potent, non-specific oxidation in alkaline conditions and high temperatures should be avoided [16,17]. Extraction should include enrichment, purification and solvent fractioning steps [16] to produce chondroitin sulfate with a narrow molecular weight range. Purification protocols are important to minimize contaminants, which can include other glycosaminoglycans, proteins, small organic molecules, viruses, prions and solvents [15,17,20,21]. Care should be taken to minimize further structural modifications during purification [15,21].

### 2.3. Regulation

Chondroitin sulfate is classified as a biological active substance by the European Medicines Agency (EMA) [22]. Only pharmaceutical-grade chondroitin sulfate is evaluated for purity, content and physico-chemical parameters using sensitive, specific, validated and published analytical approaches [15,17]. However, nutraceutical-grade chondroitin sulfate is not subject to such strict regulations.

### 2.4. Content

A US study by Adebowale *et al*. [23] has compared the amount of chondroitin sulfate in 11 different formulations against a chondroitin sulfate considered to be a chemical reference substance by the European Pharmacopeia [17]. The latter is of bovine trachea origin and manufactured by Bioibérica (Barcelona, Spain). High performance liquid chromatography (HPLC) data showed that the amount of chondroitin sulfate varied from approximately 10% to 110% of that claimed on the label [23]. Moreover, using a titration method, the amount of chondroitin sulfate in 32 products purchased from pharmacies and health food stores varied from 0% to 105% of the label claim (26/32 contained <90% label claim and 17/32 contained <40% label claim) [23].

### 2.5. Composition and Chemical Properties

A study by Volpi *et al*. [17] analyzed the composition of chondroitin sulfate from bovine, porcine, galline (chicken) and piscine (shark and skate) cartilage. Molecular weights for bovine, porcine and chicken chondroitin sulfates (14–26 kDa) were similar to the reference standard as described above (21.4 kDa), but those for shark and skate chondroitin sulfates (50–70 kDa) were considerably higher [17]. Not surprisingly, the bovine sample had similar proportions of chondroitin-4-sulfate (61%) and chondroitin-6-sulfate (33%) as the standard (61% and 34%, respectively), but the chicken and porcine samples had higher levels of chondroitin-4-sulfate (72% and 80%, respectively), while the skate and shark samples had higher levels of chondroitin-6-sulfate (39% and 50%, respectively). Also, the skate and shark chondroitin sulfates had 15% and18% chondroitin disulfates, respectively, compared with 0% for the other samples and the standard, which elevated their charge density (1.08–1.20 *versus* 0.90–0.96) [17].

A study by Tat *et al*. [16] compared three formulations of chondroitin sulfate—named CS1, CS2 and CS3—from different origins. As illustrated in Table 1, chondroitin sulfate content, molecular weight, protein content and sulfation varied between these products. Of note, CS1 had the most free sulfates (0.75%) and the lowest molecular weight, which suggests more desulfation and depolymerization during manufacturing.

Similar discrepancies in chondroitin sulfate composition have also been reported in a study from Sakai *et al*. [24] who analyzed the composition of 12 chondroitin sulfates of nutraceutical grade and found a large variation in disaccharide content: non-sulfated chondroitin 1%–9%, chondroitin-4-sulfate 26%–70%, chondroitin-6-sulfate 23%–63%, chondroitin-2,6-disulfate 0%–13% and chondroitin-4,6-disulfate 0%–3% [24].

Another study [25], which used capillary electrophoresis to quantify chondroitin sulfate concentrations, reported a large variation in disaccharide content of 11 commercially available formulations: non-sulfated chondroitin 2%–6%, chondroitin-4-sulfate 23%–85%, chondroitin-6-sulfate 11%–58%, chondroitin-2,4-disulfate 0%–8%, chondroitin-2,6-disulfate 0%–5%, chondroitin-4,6-disulfate 0%–13% and hyaluronic acid 0%–1.5%. This resulted in charge densities of 0.95 to 1.07.

**Table 1:** Chondroitin sulfate characteristics. Reproduced with permission from Tat, *et al*. [16].

Characteristic	CS1	CS2	CS3
Species	Porcine	Bovine	Bovine
Chondroitin sulfate content (%)	90.4	96.2	99.9
Molecular weight ^a^ (kDa)	12.9	13.8	15.1
Protein (%)	7.4	3.3	ND
Intrinsic viscosity (m^3^/kg)	0.034	0.036	0.040
Chlorides (%)	0.70	0.02	0.34
Free sulfates (%)	0.75	0.05	0.14
Oxalate (%)	0.021	ND	0.01
Sodium (%)	7.10	6.75	7.05
Non-sulfated chondroitin (%)	5.9	5.1	5.7
Chondroitin-4-sulfate (%)	78.3	72.7	62.8
Chondroitin-6-sulfate (%)	15.8	21.3	31.5
Chondroitin-2,6-disulfate (%)	ND	0.4	ND
Chondroitin-4,6-disulfate (%)	ND	0.5	ND

^a^: Indicates average molecular weight. CS, chondroitin sulfate; ND, not detected.

### 2.6. Absorption, Bioavailability and Bioequivalence

Adebowale *et al*. [23] studied the permeability of chondroitin sulfate across Caco-2 cell monolayers as an indicator of intestinal drug absorption. They found that permeability coefficients increased (101, 125 and 162 nm/s) with decreasing molecular weight (16.9, 8.0 and 4.0 kDa, respectively), predicting that lower molecular weight chondroitin sulfate might be more readily absorbed and hence more effective. The permeability coefficients of five other chondroitin sulfate samples (of unknown molecular weights) were lower than the ones above and ranged from 0 to 87 nm/s.

Determining the bioavailability of chondroitin sulfate is challenging but, overall, bioavailability seems to be around 10%–20% [20]. During ingestion and absorption, it is partly degraded, resulting in a variety of molecular weight metabolites in the plasma [26,27,28]. This makes it difficult to establish bioequivalence from concentration–time curves [29,30]. However, one bioequivalence method could be the use of the time course of pharmacological response using a modified Hill equation that contains terms relating to time, effect (minimum, maximum and at time T), time to achieve 50% effect and a sigmoid slope factor [29,31]. The reference standard was calculated in 24 patients in whom the chondroitin sulfate treatment continued for ≥90 days, with ≥7 determinations of effect [29]. To test another formulation and show bioequivalence, the same protocol should apply. If the 95% confidence intervals of the ratio of reference:test values are within 0.8–1.2, the formulations can be considered to be bioequivalent.

### 2.7. Biological Effects

Chondroitin sulfate has been shown to elicit a range of beneficial effects: anti-inflammatory effects, an increase in type II collagen and proteoglycans, a reduction in bone resorption and a better anabolic/catabolic balance in chondrocytes [4,19,32,33]. However, as described above, as formulations of chondroitin sulfate can vary due to their origin, production and purification, their biological effects can also differ.

Au *et al*. [34] compared the anti-inflammatory effects of 10 chondroitin sulfate products with the abovementioned reference standard. While the reference standard reduced tumour necrosis factor (TNF)-α expression in activated bovine chondrocytes by 56%, the 10 comparator products elicited 0%–53% reductions [34]. Similar discrepancies were seen in the reductions in interleukin (IL)-1β expression (60% *vs.* 0%–60%) and COX-2 gene expression (50% *vs.* 2%–52%) [34]. Results in activated THP-1 cells yielded similar data. This clearly illustrates that the effects are not consistent across formulations.

**Figure 2:**
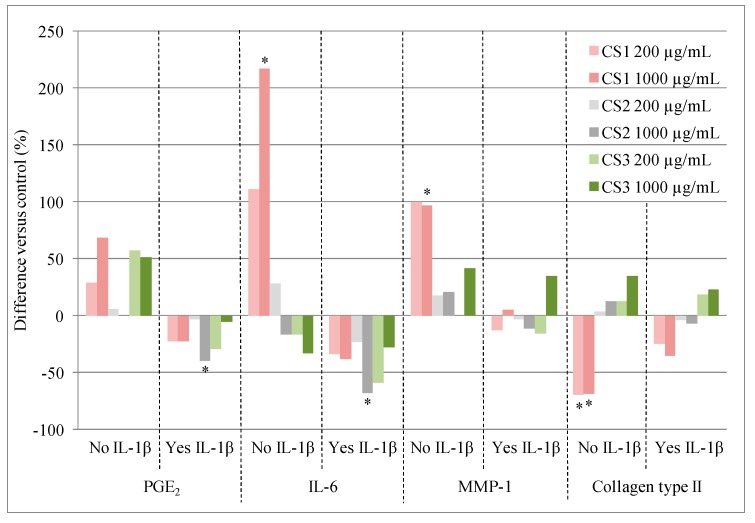
Effects of three chondroitin sulfate (CS) formulations on human osteoarthritic chondrocytes in the absence and presence of IL-1β. Adapted with permission from Tat *et al.*, * *p* < 0.03.

Tat *et al*. [16] examined the effects of two concentrations (200 and 1,000 µg/mL) of three chondroitin sulfate products (CS1, CS2 and CS3, detailed in Table 1) on human chondrocytes from the articular cartilage of patients with osteoarthritis undergoing total knee arthroplasty. Their effects on the following markers were examined: prostaglandin E_2_ (PGE_2_), an inflammatory mediator; IL-6, a pro-inflammatory cytokine; MMP-1, which leads to collagen breakdown; and collagen type II, which is a major component of cartilage. As illustrated in Figure 2, only CS1 (porcine) at 1000 µg/mL elicited significant effects in the absence of IL-1β, but these were detrimental: it increased IL-6 and MMP-1 production and decreased collagen type II expression (Figure 2). 

In the presence of IL-1β (an important pro-inflammatory mediator), which significantly increased PGE_2_, IL-6 and MMP-1 and significantly decreased collagen type II expression [16], all three chondroitin sulfates elicited beneficial responses for PGE_2_ and IL-6. These were statistically significant for inhibition of PGE_2_ and IL-6 production with 1000 µg/mL CS2 (*p* < 0.03), and there was a trend towards significance (*p* < 0.06) with 200 µg/mL CS3 (Figure 2) [16]. The authors concluded that these differences could be due to the varying amounts of chondroitin-4-sulfate and chondroitin-6-sulfate in the formulations (see Table 1) and/or differences in purity. The same three chondroitin sulfate samples (Table 1) have also been tested in a recent pharmacoproteomic study [21]. This study examined how the chondroitin sulfate formulations modulated the expression of proteins in human chondrocytes from the cartilage of patients with osteoarthritis [21]. Data agreed with the abovementioned data [16], and showed that CS1 induced a pro-inflammatory state and activated catabolic pathways (*i.e.*, molecular degradation) while CS2 and CS3 were anti-inflammatory and induced an anabolic response (*i.e.*, molecular construction) [21]. Specifically, CS1 caused an increase in the mitochondrial superoxide dismutase (SODM), the cartilage oligomeric matrix protein (COMP), as well as interstitial collagenase (MMP-1), stromelysin-1 (MMP-3), and pentraxin-related protein (PTX3) levels. On the contrary, CS2 and CS3 stimulated the expression of a number of structural proteins, growth factors, and extracellular matrix proteins. CS2 decreased the expression of galectin-3 (LEG3) and proteasome subunit β type-1 (PSB1) and CS3 was able to increase caldesmon 1 (CALD1), heat shock cognate 71-kDa protein (HSP7C), and vimentin (VIME), whereas they decreased neutral α-glucosidase AB (GANAB), nicotinamide phosphoribosyltransferase (NAMPT), glutamatedehydrogenase1, mitochondrial (DHE3), PSB1, and peroxiredoxin-4 (PRDX4).

Evidence has shown that alterations in the subchondral bone, which may occur quite early in the osteoarthritis process, are part of the initiation/pathological process. In osteoarthritis, this tissue has been shown to be the site of numerous dynamic morphological transformations resulting in an altered osteoblast metabolism which instigates increased bone resorption early on during the disease progression. Hence, osteoblasts from human osteoarthritic subchondral bone produce an excess of many biochemical factors favouring the maturation/activation of osteoclasts (cells involved in bone resorption) and/or resorption of bone matrix. Cantley *et al*. [35] compared the abilities of bovine-, porcine- and piscine-derived chondroitin sulfate to suppress osteoclast formation and activity. The chondroitin sulfate samples tested had inconsistent effects on the formation of osteoclasts [35]. However, bovine-derived chondroitin sulfate consistently suppressed osteoclast activity, and thus bone resorption, at concentrations as low as 1 µg/mL. Piscine and porcine chondroitin sulfate had less consistency in their effects [35]. The data on bovine chondroitin sulfate agree with a study in human osteoarthritic subchondral bone osteoblasts with the above-mentioned chondroitin sulfate reference standard (from bovine trachea), in which the expression of two bone biomarkers found in abnormal levels in human osteoarthritic subchondral bone osteoblasts was tested [36]. The bone biomarkers were osteoprotegerin (OPG), a factor that prevents osteoclastogenesis and thus inhibits bone resorption, and the receptor activator of nuclear factor-kappa B ligand (RANKL), an essential cytokine for osteoclast differentiation and bone loss. Data showed that this chondroitin sulfate increased the expression of OPG and decreased RANKL, thereby increasing the ratio of OPG/RANKL. Finally, a study looking at the ability of oversulfated chondroitin sulfate from various sources to regulate the differentiation of osteoclasts showed that chondroitin-4,6-disulfate (from squid cartilage) and high-dose chondroitin-2,6-disulfate (from shark cartilage) significantly reduced differentiation, while chondroitin-4-sulfate (from whale cartilage) and chondroitin-6-sulfate (from shark cartilage) did not [37].

### 2.8. Clinical Effects

Various meta-analyses, published between 2000 and 2012, have concluded that chondroitin sulfate has a beneficial effect on osteoarthritis pain, symptoms, function and radiological progression [38,39,40,41,42,43,44]. More recent studies have also reported a beneficial effect on cartilage volume and bone marrow lesions [45] and on risk of total knee replacement [46,47]. However, other meta-analyses have concluded little or no benefit of chondroitin sulfate [48,49]. This may be due, at least partly, to the inclusion of studies that used non-pharmaceutical grade chondroitin sulfate [50,51]. Pharmaceutical-grade chondroitin sulfate has been used in several published randomized double-blind clinical trials over the last 25 years [4], which have demonstrated its efficacy and safety in patients with osteoarthritis.

To identify a suitable source of chondroitin sulfate for the Glucosamine/Chondroitin Arthritis Intervention Trial (GAIT), 20 commercially available US products were considered but rejected, due to a lack of compliance with current good manufacturing practices for pharmaceuticals and issues with factors such as source, purity, content variability, inclusion of other dietary supplements of trace elements and manufacturing procedures [18]. Two of the pharmaceutical grade products were further tested against a test standard (Sigma). Based on comparative study data, the chondroitin sulfate derived from bovine trachea from Bioibérica was chosen. GAIT was a randomized, controlled study in patients with painful knee osteoarthritis [52]. Patients were randomized to chondroitin sulfate, glucosamine, chondroitin sulfate and glucosamine, celecoxib or placebo for 24 weeks. A 20% decrease in pain was achieved in 65%, 64%, 67%, 70% and 60% of patients, respectively, but this was only significantly different to placebo for celecoxib (*p* = 0.008). In a sub-group of patients with moderate-severe pain at baseline, a 20% decrease in pain was achieved in 61%, 66%, 79%, 69% and 54%, respectively, and this was found to be significant only for chondroitin sulfate and glucosamine (*p* = 0.002). However, significance may have been compromised by a number of factors, including that 79% of the patients had mild pain, there was a high placebo response rate, rescue medication with high doses of paracetamol was allowed, and about 20% of patients did not complete the study [51].

### 2.9. Safety

Chondroitin sulfate has demonstrated an excellent safety profile [52,53]. However, it is very important to use a pharmaceutical-grade product to avoid any safety issues, since it is extracted from biological origin and any changes in its physico-chemical properties could modify its safety profile. It is also possible that some products could be contaminated by other glycosaminoglycans, proteins, small organic molecules, viruses, prions and solvents [15,17,20,21]. Of note, serious adverse events (including acute hypersensitivity and death) have been reported due to contaminated heparin [54,55], which is also extracted from biological sources.

## 3. Conclusions

Chondroitin sulfate products can be derived from a range of animal tissues, which could lead to products having different structures; and a variety of extraction and purification techniques can be used, resulting in different content, composition, purity, biological effects, clinical efficacy and safety. Therefore, chondroitin sulfate product quality should be regulated and standardized [15,17,51].
